# A Case Report Depicting a Rare Neurosurgical Disease: Aggressive Meningiomatosis

**DOI:** 10.3390/jcm14082731

**Published:** 2025-04-16

**Authors:** Ligia Gabriela Tataranu

**Affiliations:** 1 Department of Neurosurgery, Carol Davila University of Medicine and Pharmacy, 020021 Bucharest, Romania; ligia.tataranu@umfcd.ro; 2 Department of Neurosurgery, Bagdasar-Arseni Emergency Clinical Hospital, 041915 Bucharest, Romania

**Keywords:** meningioma, aggressive meningiomatosis, meningiomatosis, multiple meningiomas

## Abstract

**Background**: Although meningiomas are typically solitary lesions, occasionally, two or more separate tumors can occur simultaneously or sequentially, in which case the terms “multiple meningiomas” (MM) or “meningiomatosis” are used. Aggressive meningiomatosis is a rare entity that can significantly influence survival rates and quality of life. **Methods**: The current article aims to report an interesting case of a 54-year-old Caucasian woman with aggressive meningiomatosis and no relevant familial history. The patient had a history of a left convexity frontal meningioma, resected in October 2023 and identified as a meningothelial meningioma, followed by a left convexity frontopolar meningioma, resected in May 2024 and identified as an anaplastic meningioma. Furthermore, while the first lesion rapidly recurred, an important change in the histopathological grade was observed, and a diagnosis of aggressive meningiomatosis was established. **Results**: The particularity of this case is given not only by the aggressive growth pattern but also by the different histopathological gradings of the meningiomas and the anaplastic transformation of the recurrence. **Conclusions**: Aggressive meningiomatosis is a challenging medical condition for which rigorous follow-up is mandatory throughout the lifespan. New tumors with different gradings and localizations can arise, and each must be treated as a new entity. The lack of therapeutic protocols in MM makes such case reports valuable, as they highlight the necessity of specific therapeutic recommendations.

## 1. Introduction

Originating from the arachnoid cap cells, meningiomas represent around one-third of primary intracranial tumors in adults and are most commonly solitary tumors [1,2]. Nevertheless, the presence of at least two lesions with different intracranial locations that appear synchronous or metachronous has been defined as multiple meningiomas (MM) or meningiomatosis [3]. The first description of MM was in 1889 by Anfimow and Blumenau; however, in 1938, Cushing and Eisenhardt were the first to attempt to delineate meningiomatosis as a separate clinical entity, and nowadays it is considered a distinct clinical condition [4,5,6,7]. Occasionally, different tumoral grading has been identified.

In the current fifth edition (2021) of the World Health Organization (WHO) Classification of Tumours of the Central Nervous System, meningioma is considered a single type, with 15 histopathologic subtypes. Based on clinicopathological aspects, meningiomas are classified as grade 1 (benign), grade 2 (atypical), and grade 3 (anaplastic) [8]. The vast majority of meningiomas are diagnosed as WHO grade 1 and have a low recurrence rate and a good prognosis in comparison to higher grades, which have a higher risk of recurrence and a lower survival rate [8,9]. However, the WHO grade alone is not sufficient to estimate the prognosis. Therefore, several molecular biomarkers are already being used to achieve a complete diagnosis to differentiate between meningiomas and other meningeal tumors, such as solitary fibrous tumors (SFTs), mainly to guide the selection of optimal therapies for disease control [8,10].

The epidemiological data regarding MM conclude that up to 10% of all meningioma cases are affected, although recent data indicates a higher incidence [7]. Furthermore, it has been stated that MM is more predominant in female patients [11]. A brief categorization based on etiological factors divides patients with MM into sporadic, familial, and radiation-induced cases [11].

The therapeutic management is similar to solitary meningioma, as each tumor is treated individually considering size, location, and general neurological condition [12]. Notwithstanding the fact that treatment for meningiomas comprises an intricate approach, the first choice in current standard care is neurosurgical excision through gross total resection (GTR) if the tumor is surgically accessible, voluminous, and with peritumoral edema, and if the patient is young and in good clinical condition [7]. The goals of the tumoral resection are to relieve the neurological symptoms caused by the mass effect and to sample tissue for diagnosis [13,14]. In addition, GTR classified as Simpson 1 to 3 has been historically considered a major predictor of recurrence. The neurosurgical choice of GTR is still controversial, given that a maximal safe resection (MSR) is preferred over an aggressive resection, which carries increasing morbidity [13,15].

Besides surgery, available therapeutic approaches comprise stereotactic radiosurgery and fractionated external beam radiotherapy. These options are taken into consideration in tumors with surgically unfavorable locations, in older patients in poor clinical condition, as well as in cases of multiple tumors requiring treatment [7]. Occasionally, the therapeutic approaches can be combined, depending on individual factors [7].

The recurrence rates in MM vary from 13% to 29% [16], with an average growth rate of 0.46 cm^3^/year (range 0.57–2.94 cm^3^/year) [7].

It is worth mentioning that, despite the presence of multiple meningiomas in the same patient, the prognosis is similar to solitary meningiomas, even if various grades of malignancy can be observed in one-third of MM patients [4,17].

The current case report reviews a distinctive case of MM, with particularity given not only by the aggressive growth pattern but also by the different histopathological gradings of the meningiomas and the anaplastic transformation of the recurrence. The presented article aims to add further information to the scarce literature regarding the optimal treatment and follow-up for MM patients and the particular challenges related to the number of tumors and the possibility of malignant transformation, even in WHO grade 1 tumors. Moreover, this article highlights the lack of specific therapeutic protocols in MM and the necessity of particular management recommendations, as is the case in solitary meningiomas.

## 2. Case Presentation

### 2.1. The Onset of an Intricate Personal History and the First Surgery

**Figure 1:**
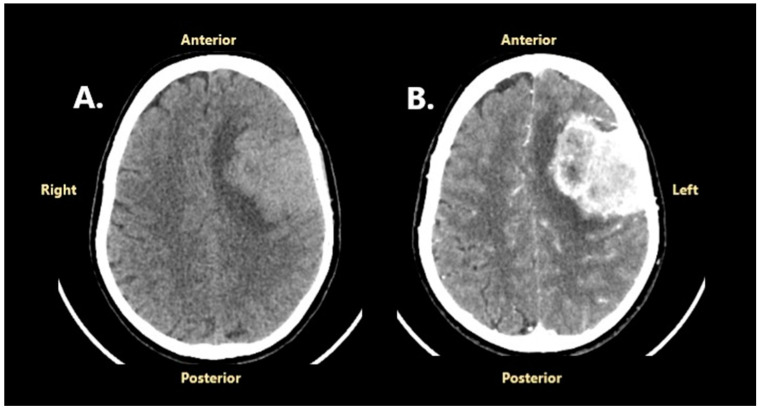
Preoperative cranial CT scan performed in October 2023: (**A**)—without contrast enhancement; (**B**)—with contrast enhancement.

A 53-year-old Caucasian woman with no significant personal or familial medical history and no prior radiation exposure was admitted to the neurosurgical department of another hospital in October 2023 for headache, expressive aphasia, tonic–clonic seizures, and predominant upper limb right-sided hemiparesis. A cranial CT scan showed an extra-axial left convexity frontal mass lesion with a broad dural base, well-circumscribed and polylobate, with an anteroposterior diameter of 62 mm, a transversal diameter of 52 mm, and a craniocaudal diameter of 55 mm. The lesion was moderately hyperdense compared to normal brain tissue on a non-contrast CT scan and showed high contrast enhancement on the post-contrast CT scan, which highlights small hypodense areas (cystic degeneration or necrosis). An important perilesional edema and a midline shift of 12 mm were also revealed (Figure 1).

A neurosurgical intervention (using a frontoparietal approach), with GTR and a coagulation of dural attachments (Simpson grade II), without intraoperative or postoperative complications, was achieved. The tumor sample was sent for histopathological examination, establishing that the tumor was a meningothelial meningioma, WHO grade 1, with small necrosis areas, rare psammomatous bodies, and mitotic activity of 4 mitoses per 10 high-power fields (HPF) in a possible hot spot. Immunohistochemistry (IHC) indicated the following results: Somatostatin Receptor 2 (SSTR2)—diffuse positive; Signal Transducer and Activator of Transcription 6 (STAT-6)—negative; Progesterone Receptor (PGR)—negative; Phosphorylated Histone H3 (PHH3)—positive in 5 mitoses/10 HPF; and the Ki67 proliferation index was 25% (Table 1).

**Figure 2:**
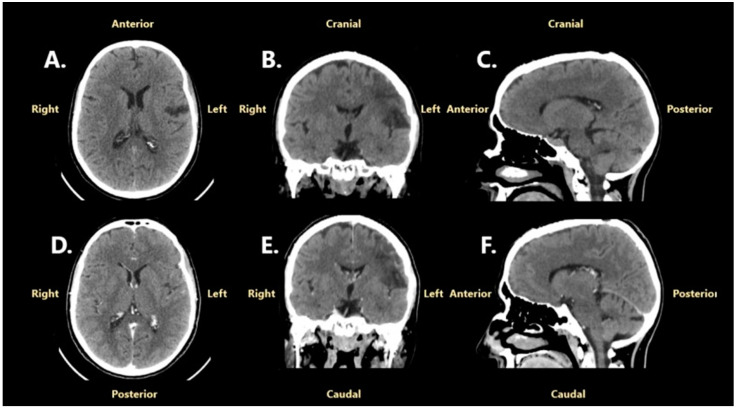
A cranial CT scan performed in December 2023, 3 months after the neurosurgical intervention: (**A**)—non-contrast, axial projection; (**B**)—contrast enhancement, coronal projection; (**C**)—non-contrast, sagittal projection; (**D**)—contrast enhancement, axial projection; (**E**)—contrast enhancement, coronal reconstruction; (**F**) contrast enhancement, sagittal reconstruction.

Following surgery, the patient went into progressive remission of neurological symptoms and fully recovered after 3 months. In December 2023, at the 3-month evaluation, a cranial CT scan of the brain was performed, which confirmed gross total resection. Furthermore, no sign of tumor recurrence was noted (Figure 2). The patient was informed about the neurosurgical status and was advised for a reevaluation after 6 months.

### 2.2. Admission to Our Neurosurgical Department and the Second Surgery

**Figure 3:**
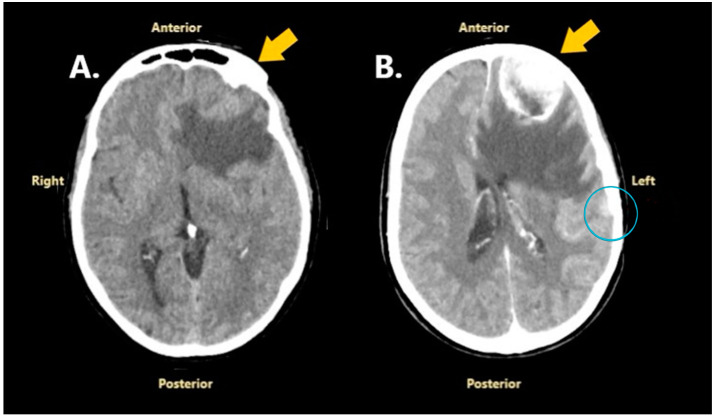
A cranial CT scan performed in May 2024: (**A**)—without contrast enhancement; (**B**)—with contrast enhancement. The yellow arrows point to the left frontopolar meningioma, and the blue circle surrounds the left frontoparietal recurrent lesion.

In May 2024, 6 months after the initial surgery, the patient developed a severe headache, expressive aphasia, and predominant upper limb right-sided hemiparesis. This time, she was admitted to our hospital, the Neurosurgical Department of Bagdasar-Arseni Clinical Emergency Hospital, for further investigations and treatment. A cranial CT scan revealed a new dural-based left frontopolar mass with well-defined margins, spontaneously isodense to gray matter, and with heterogenous enhancement after contrast administration. The lesion had diffuse perilesional edema, a midline shift of 13 mm, and subfalcine herniation. Moreover, another left frontoparietal nodular lesion with similar features was observed under the bone flap of the initial craniotomy (Figure 3). The CT scan was suggestive of meningiomas for both lesions.

**Figure 4:**
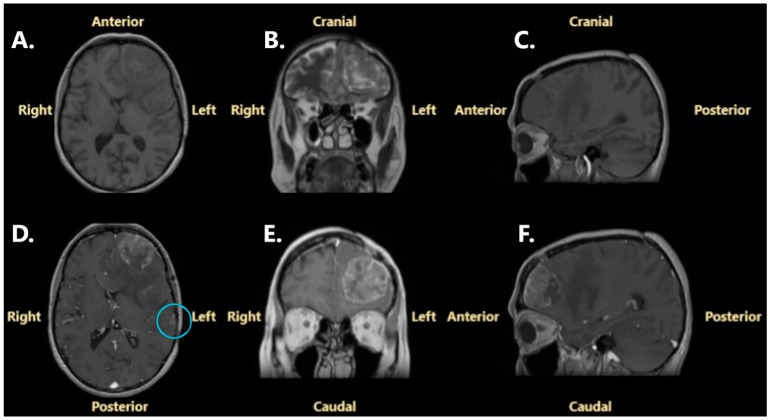
Brain MRI performed in May 2024: (**A**)—non-contrast T1-weighted Fast Spoiled Gradient-Echo (FSPGR) sequence, axial projection; (**B**)—non-contrast T2-weighted Fast Spin Echo (FSE) sequence, coronal projection; (**C**)—non-contrast T1-weighted Multiplanar Reconstruction (MPR), sagittal projection; (**D**)—contrast enhancement on T1-weighted FSPGR, axial projection; (**E**)—contrast enhancement on T1-weighted MPR T1, coronal projection; (**F**)—contrast enhancement on T1-weighted MPR, sagittal projection. The blue circle surrounds the left frontoparietal extra-axial tumor.

A further brain MRI was performed, revealing in detail the left frontopolar mass, measuring 45 mm/37 mm/42 mm (anteroposterior/transversal/craniocaudal diameters), and the left frontoparietal mass, with dimensions of 13 mm/17 mm/10 mm (anteroposterior/transversal/craniocaudal diameters). The new left frontopolar mass appeared as isointense on T1-weighted sequences, isointense to hyperintense on T2-weighted sequences, hyperintense on FLAIR sequences, and showed high contrast enhancement. A heterogenous enhancement pattern and severe vasogenic edema in adjacent brain parenchyma were also demonstrated (Figure 4).

These neuroimaging findings indicated a new left convexity frontopolar tumor, with typical characteristics of a meningioma, and a recurrence of the previously operated left convexity frontal meningioma.

After the preoperative evaluation, a neurosurgical intervention was decided upon, and the patient gave her written consent. Thus, in May 2024, a gross total resection (Simpson grade II) was performed through a transcranial left frontal approach under general anesthesia. During the operation, neighboring the inferior frontal gyrus, a reddish inhomogeneous lesion was found, with a mixed consistency given by the cystic, hemorrhagic, and necrotic components. The tumoral mass had clear boundaries, and the macroscopic aspect was typical of a meningioma.

### 2.3. Histopathological and Immunohistochemical Study After the Second Surgery

**Figure 5:**
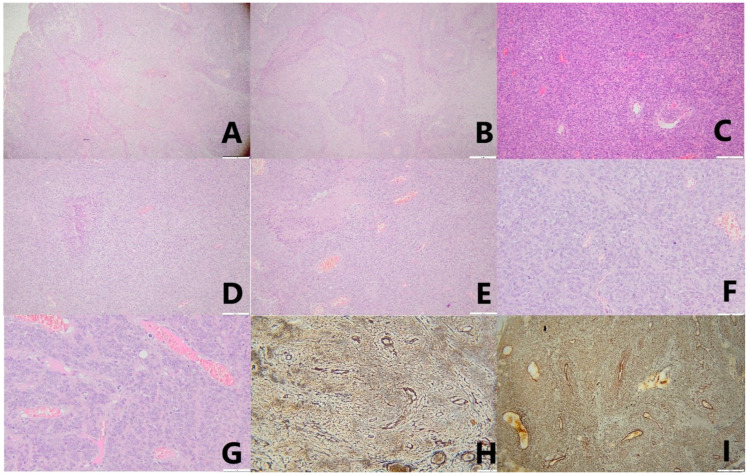
Photomicrographs depicting the histopathologic features of the left frontopolar meningioma. (**A**)—hematoxylin–eosin stain (H&E), magnification 40×: cytoarchitecture; (**B**)—H&E stain, magnification 10×: geographic necrosis; (**C**)—H&E stain, magnification 40×: intense mitotic activity and blood vessels; (**D**)—H&E stain, magnification 20×: loss of cellular architecture, increased number of mitoses, and intense vascularization; (**E**)—H&E stain, magnification 20×: abnormal cytoarchitecture and intense vascularization; (**F**,**G**)—H&E stain, magnification 40×: intense mitotic activity; (**H**)—reticulin stain, magnification 20×: dense reticulin network; (**I**)—reticulin stain, magnification 10×: dense reticulin network.

The pathological examination revealed anaplastic meningioma (WHO grade 3), with tumoral mesenchymal proliferation, nuclear pleomorphism, important mitotic activity (>20 mitoses/10 HPF), vascular slits at the level of the stroma, geographic necrosis, and a dense reticulin network, shown when using a special coloring for reticulin (Figure 5).

Following the pathological examination, IHC was performed to achieve a differential diagnosis with SFT, assess the proliferative markers (Ki67), and obtain more information about the tumoral status and aggressiveness. The SSTR2 marker was diffuse positive, STAT6 negative, PGR negative, and PHH3 positive in 20 mitosis/10 HPF 40×, and the Ki67 proliferation index was 50% (Table 2).

Notable differences were observed in the biological profiles of the two meningiomas. The second meningioma showed evidence of a more aggressive tumor, given the increase in the Ki67 index from 25% to 50%, the presence of geographic necrosis, and the increase in the mitotic activity from 4 mitoses/10 HPF to more than 20 mitoses/10 HPF.

### 2.4. Postoperative Course and Follow-Up

**Figure 6:**
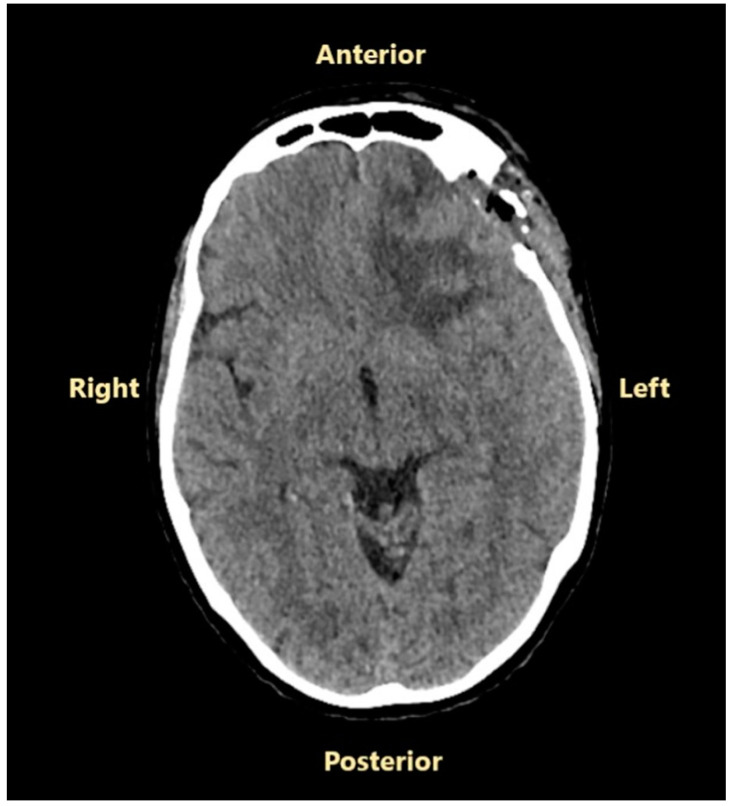
Postoperative native cranial CT scan, performed in May 2024.

Following surgery, the patient had a partial recovery of neurological symptoms. Thus, the headache disappeared, and the right hemiparesis fully recovered. However, elements of expressive aphasia were still present. Two days after the surgery, a postoperative native cranial CT scan showed normal postoperative findings (Figure 6).

**Figure 7:**
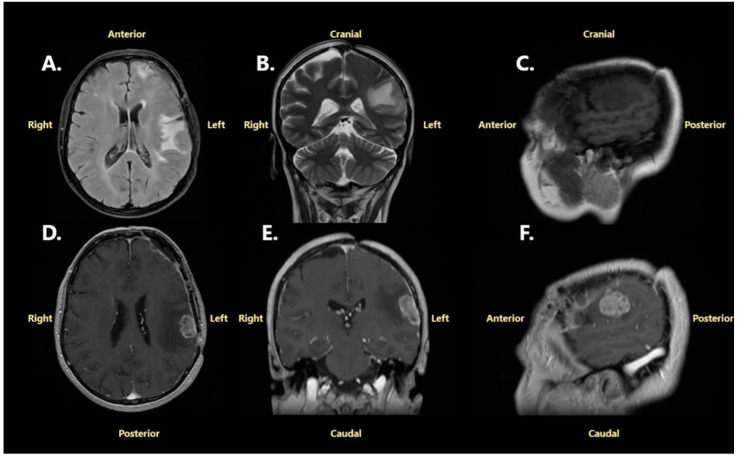
Brain MRI performed in July 2024: (**A**)—fluid-attenuated inversion recovery with fat saturation (FLAIR FS) T2-weighted sequence, axial view; (**B**)—T2 FSE sequence, coronal projection; (**C**)—T1-weighted image, sagittal projection; (**D**)—T1-weighted MPR sequence with contrast enhancement, axial projection; (**E**)—T1-weighted MPR sequence with contrast enhancement, coronal projection; (**F**)—T1-weighted MPR sequence with contrast enhancement, sagittal projection.

One month after the surgery, the patient’s clinical status remained unchanged. Still, a cranial MRI revealed a growth of the left frontoparietal tumor recurrence, which measured 25 mm/15 mm/15 mm (anteroposterior/transversal/craniocaudal diameters) (Figure 7). Considering the recommendations of the European Association of Neuro-Oncology [18], a clinical and neuroimaging evaluation after three months was advised to observe the tumoral progression of the left frontoparietal meningioma recurrence and to assess the possibility of reintervention.

### 2.5. The Tumoral Recurrence Associated with the First Surgery

**Figure 8:**
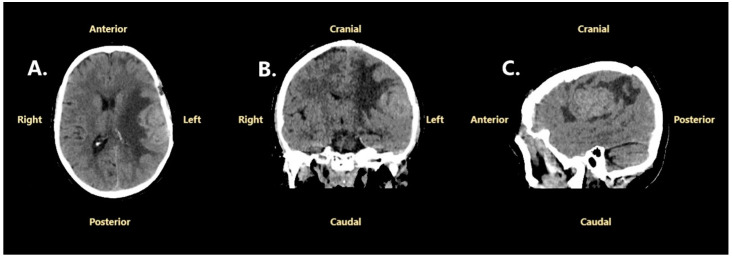
Native cranial CT scan performed in August 2024: (**A**)—axial projection; (**B**)—coronal projection; (**C**)—sagittal projection.

The patient was readmitted to our hospital in August 2024, two months after the first follow-up, for severe headache, tonic–clonic seizures, expressive and receptive aphasia, and right central facial palsy. A native cranial CT scan revealed an important progression of the left frontoparietal tumor recurrence, which appeared moderately hyperdense compared to normal brain tissue, with significant adjacent edema (Figure 8).

**Figure 9:**
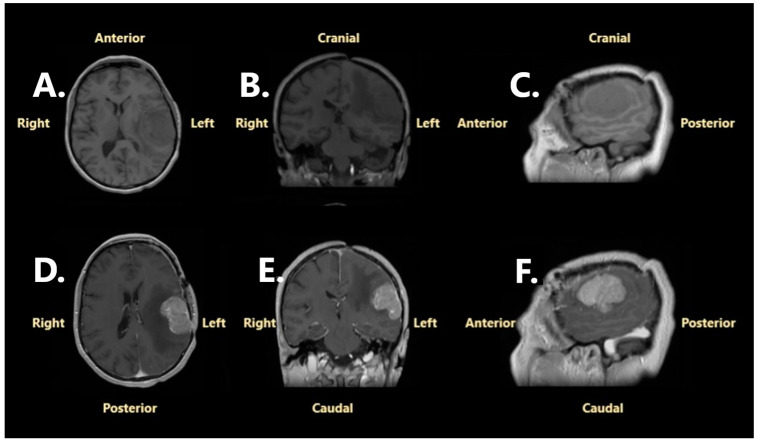
Brain MRI performed in August 2024; (**A**)—native T1-weighted MPR sequence, axial projection; (**B**)—native T1-weighted MPR sequence, coronal projection; (**C**)—native T1-weighted MPR sequence, sagittal projection; (**D**)—T2-weighted FLAIR FS sequence, axial projection; (**E**)—T2-weighted FLAIR FS sequence, coronal projection; (**F**)—3D T2 FLAIR FS sequence, sagittal projection.

A subsequent brain MRI showed a polylobate mass lesion with dural attachment, isointense relative to gray matter on the T1-weighted and T2-weighted sequences, intense and relatively homogenous enhancement, and extensive peritumoral edema. The tumor measured 46 mm/42 mm/40 mm (anteroposterior/transversal/craniocaudal diameters) and caused a 5 mm midline shift (Figure 9).

After evaluating all of the neuroimaging and neurological results, a third neurosurgical intervention was decided upon in August 2024 and approved by the patient. Consequently, a left frontoparietal approach with temporal extension was performed on the old surgical site. After the craniotomy, a moderately inhomogeneous yellowish-white lesion with inferior dural insertion was discovered. This time, A GTR was achieved as a Simpson grade 1, with the complete removal of the affected dura mater. It is worth mentioning that after close inspection, it was concluded that there was no bone infiltration.

Although the headache went into remission, and the facial palsy, the seizures, and the receptive aphasia fully recovered, elements of expressive aphasia remained.

**Figure 10:**
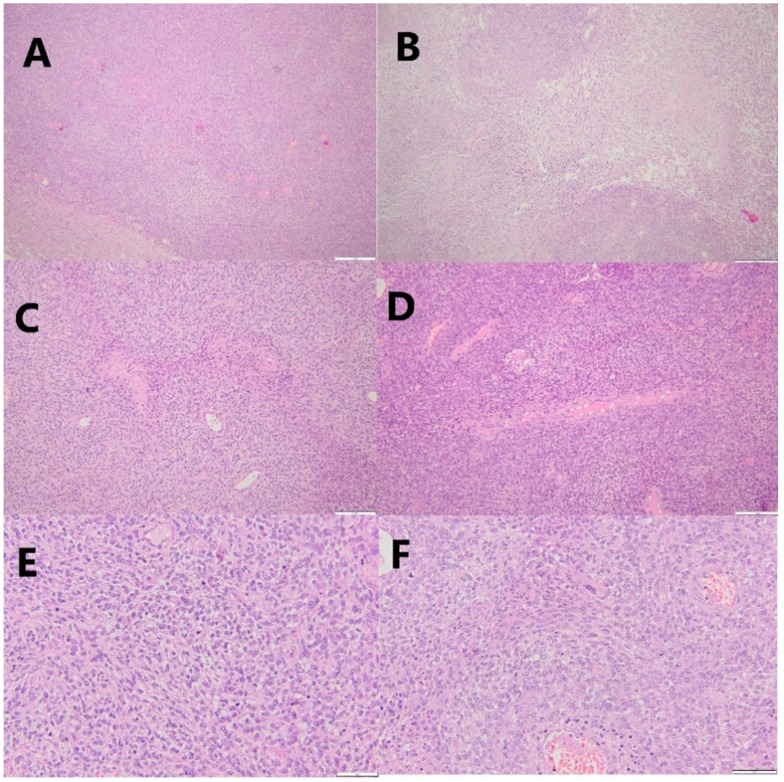
Photomicrographs depicting histopathologic features of the left frontoparietal recurrence of meningioma. (**A**)—H&E stain, magnification 10×: invasion of the meningioma in the cerebral tissue; (**B**)—H&E stain, magnification 10×: geographic necrosis; (**C**)—H&E stain, magnification 20×: cells with important nuclear atypia, big eosinophilic nucleoli; (**D**)—H&E stain, magnification 20×: cells with important nuclear atypia, big eosinophilic nucleoli; (**E**)—H&E stain, magnification 40×: frequent mitoses—more than 20 mitoses/10 HPF; (**F**)—H&E stain, magnification 40×: vascular proliferation and intense mitotic activity.

Histopathological examination recorded the heterogenous appearance of the tumoral tissue sampling, with necrosis and hemorrhagic spots. Microscopic examination revealed tumoral cells with important nuclear atypia, big eosinophilic nucleoli, frequent mitoses (more than 20 mitoses/10 HPF), and geographic necrosis. These characteristics were compatible with an anaplastic meningioma WHO grade 3 (Figure 10).

Following the pathological examination, IHC established the diagnosis of a high-grade meningioma, given the high histological aggressivity. The SSTR 2 marker was positive, STAT6 was negative, PGR was negative, PHH3 was positive in 20 mitoses/10 HPF 40×, and the Ki67 proliferation index was 40% (Table 3).

Even though the patient had a good neurological recovery with no perioperative complications, the increase in aggressiveness suggested a poor long-term prognosis, and the patient was referred to perform adjuvant radiotherapy to obtain better local control, increase the disease-free interval, maintain quality of life, and prolong survival.

**Figure 11:**
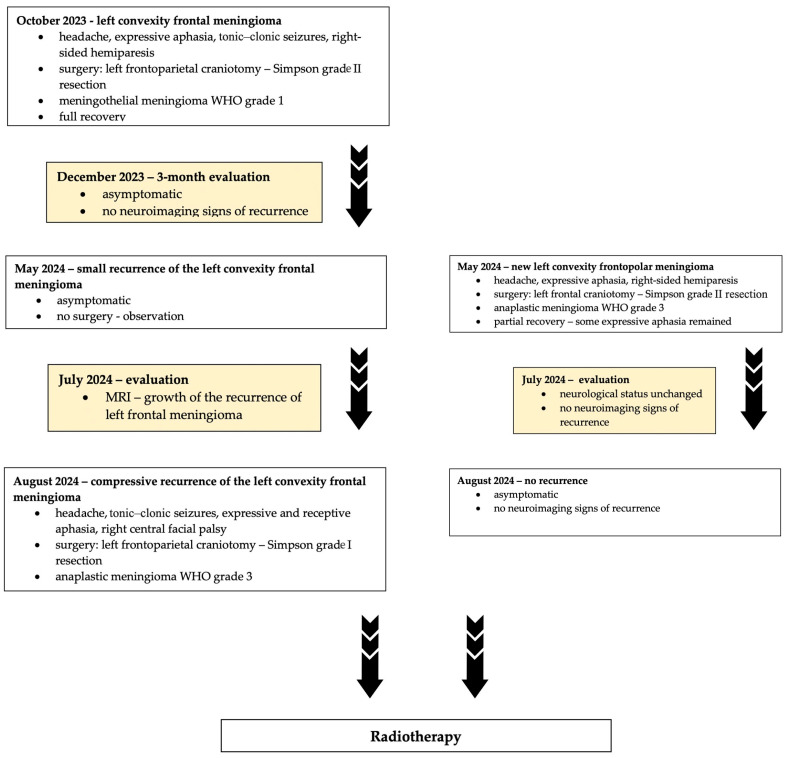
The timeline of events in the present case report.

The summary of the entire case report is illustrated in Figure 11.

## 3. Discussion

Meningiomas are usually benign, slow-growing tumors, and in some types (e.g., convexity meningiomas), a gross total resection can be easily achieved [19]. However, MM and different histological-grade meningiomas in the same patient can be especially challenging [11]. Different localizations and histological grades of tumors in MM can affect progression, prognosis and, consequently, therapeutic management. Despite its benign character, occasionally, the biological pattern of these tumors can be aggressive [3,7].

While the pathophysiology of MM is still unclear, the multicentric and monoclonal theories are primarily implicated [20]. An argument for the monoclonal theory is supported by the fact that, in MM, the most common location for meningiomas is along the convexity, with a predisposition for the same hemisphere. A hypothesis for this type of localization is its possible development from a unique monoclonal proliferation, which then spreads within the same hemisphere with the support of cerebrospinal fluid (CSF) [5,21,22]. The multicentric theory presumes that a tumor-producing factor stimulates the meningeal cells, and the tumors arise independently [6]. An argument for this theory is supported by the possibility of producing MM in mice models exposed to ethyl nitrosourea (ENU) [23].

Familial MM can be associated with genetic syndromes such as Neurofibromatosis type 2 (NF-2), Cowden syndrome, Multiple Endocrine Neoplasia 1 (MEN1), Werner syndrome, Rubinstein–Taybi syndrome, Gorlin syndrome, and Li–Fraumeni syndrome [7,11,24,25,26,27,28,29,30,31] (Table 4). In some cases, germline mutations in genes are thought to be related to meningioma initiation and progression, e.g., in the SMARCB1 and SMARCE1 mutations [32,33,34,35]. However, notwithstanding these theories, further studies are needed to establish the cause of MM and its risk factors in order to determine if triggers may be involved in the sudden increase in aggressiveness in meningiomas. Nevertheless, in the presented case, the patient had no significant family history, no prior exposure to radiation, and no comorbidities.

The differential diagnosis of MM can be challenging, given that the neuroimaging features are similar to those of other diseases. The first differential diagnosis considered is leptomeningeal metastases, which, if left untreated, can significantly decrease survival rate. The contrast-enhanced fast FLAIR sequence is more sensitive for the assessment of this pathology, not only due to the suppression of adjacent CSF but also the lack of enhancement on cortical vascularity and the magnetization transfer saturation effect [36]. Furthermore, despite similar general characteristics, leptomeningeal metastases are differentiated by histopathological examination, and they are more necrotic, with more frequent intralesional hemorrhages [37].

Secondly, a differential diagnosis can be made with SFT, an aggressive neoplasm, which can also arise from the arachnoid cap cells. This tumor can easily metastasize extracranially, while the neuroimaging aspect is very similar to MM. However, recent research demonstrated that radiomics methods can identify specific tumoral textures based on conventional MRI sequence images, leading to a simplification of the diagnosis process [38].

Another possible differential diagnosis can be made with gliomas extended into the subarachnoid space. Malignant gliomas can mimic meningiomas in neuroimaging studies, showing dural tail, cerebrospinal fluid cleft sign, large dural contact, and vascular feeders, which are usually correlated with meningiomas [39].

Infrequently, a differential diagnosis can be made with inflammatory diseases such as rheumatoid arthritis, Wegener’s granulomatosis, and extra-axial neurosarcoidosis, or even with infectious diseases such as tuberculosis or syphilitic gumma [40]. Hematopoietic neoplasms, such as extra-axial non-Hodgkin lymphoma and primary Hodgkin disease, can show similar radiological characteristics. Furthermore, rare clinical entities, such as Castleman disease, a lymphoproliferative disorder, may radiologically mimic meningioma and should be considered in the differential diagnosis, as reported by Moguel et al. [41]. IHC provides the most significant contribution to the differential diagnosis. Tumoral tissue exhibits specific markers, such as Cluster of Differentiation (CD) markers, including CD20, CD3, and CD16, in patients with Castleman disease [41]. Thus, the diagnosis can be established based on specific biomarkers.

It is worth mentioning that the pathological diagnosis plays a pivotal role in establishing the final diagnosis. Furthermore, in MM, the grading of the tumor may vary, as illustrated in the presented case. Histological examination with H&E is usually sufficient to obtain a definitive diagnosis for a resected meningioma due to typical features, such as vesiculous nuclei with prominent nucleoli and nuclear pseudo-inclusions, the presence of “psammoma bodies”, whorls of tumoral cells, the presence of mitoses, small cell formation, brain infiltration, sheeting, increased fibrosis, widespread collagen formation, and necrotic areas [40,42]. Occasionally, histopathological similarities with SFT (formerly named hemangiopericytoma) can be present. Thus, further testing is needed to obtain a definitive diagnosis. The most helpful tool in differential diagnosis and the evaluation of the tumoral profile is IHC, which for SSTR2a is considered a sensitive diagnostic marker for meningiomas [40]. SFT expresses STAT6, while meningiomas are STAT6-negative [43]. In the current case, the left frontopolar meningioma, as shown with H&E staining, revealed tumoral mesenchymal proliferation, important nuclear pleomorphism, mitotic activity (>20 mitoses/10 HPF), the presence of vascular slits, geographic necrosis, and a dense reticulin network. Features such as necrosis, high mitotic count, hypercellularity, cell atypia with round cells, and nuclear pleomorphism are also associated with SFT [44]. IHC was performed, and the diagnosis of anaplastic meningioma was obtained, the left frontopolar lesion being STAT6 negative and SSTR2a positive.

The presence of two meningiomas with different histopathological grades or patterns in the same patient is a significant aspect of clinical neuro-oncology. This occurrence may reflect more complex tumor characteristics, leading to special clinical behaviors and different treatment responses. In cases where patients harbor both grade I meningiomas and higher-grade lesions, such as grade II or grade III tumors, the treatment strategy might necessitate a more aggressive approach with adjuvant therapies for the atypical meningioma, in contrast to the potentially suitable management for its grade I counterpart. The literature, including reports such as those by Saranraj et al. and Liu et al., document rare instances of dual-grade meningiomas (a WHO grade 1 transitional meningioma and a WHO grade 1 angiomatous meningioma, or the association of a fibrous meningioma, WHO grade 1, with an atypical meningioma, WHO grade 2), drawing attention to their coexistence and the implications thereof [45,46]. These cases illustrate the need for detailed histopathological assessment, as it can significantly influence prognosis and the likelihood of tumor recurrence.

Another helpful piece of information that can be obtained with the help of IHC is represented by the Ki67 proliferation index, a nuclear protein studied as a cell proliferation marker in different types of cancer. Thus, the Ki67 index is an important predictive marker for recurrence and aggressiveness, particularly in WHO grade 2 and 3 meningiomas [47]. In the presented case, the Ki67 index was 25% for the left frontal meningioma (WHO grade 1) and 50% for the left frontopolar meningioma (WHO grade 3), the latter being more aggressive. However, ten months after surgery, the patient underwent a new surgical intervention after developing new symptomatology associated with the recurrence of the left frontal meningioma (WHO grade 1). Furthermore, the histopathological examination confirmed a shift in tumoral aggressiveness, as the meningioma transformed into a WHO grade 3 anaplastic meningioma. Malignant meningiomas are a rare entity, representing less than 1% of all meningiomas and having an overall survival rate of approximately 2–3 years [48].

The therapeutic approach for meningiomatosis is multimodal, while neurosurgical intervention is the cornerstone. The main therapeutic goal remains maximum safe resection, which is the most efficient way to achieve the relief of neurological symptoms and the mass effect, improve neurological status, prolong the disease-free interval (DFI), and maintain good quality of life [49]. Furthermore, surgery can provide tissue for histological diagnosis and IHC. The challenge in achieving disease control lies in is the risk of developing new meningiomas in technically challenging locations and the possibility of local recurrence. GTR can be easily achieved in convexity meningiomas, reducing the recurrence risk and prolonging DFI. Nevertheless, partial resection can be an option in difficult locations, and adjuvant therapies such as radiotherapy, stereotactic radiosurgery, and fractionated external beam radiotherapy can be useful [49]. Radiotherapy serves as a valuable adjuvant therapy for achieving local control and reducing the recurrence rates of anaplastic meningiomas, although its impact on overall survival remains a topic of debate [50,51]. Systemic treatment is not currently available, but clinical trials are underway for immunotherapy and targeted therapy (e.g., multi-tyrosine kinase inhibitors and vascular endothelial growth factors VEGF antibodies) [52,53]. Even with the current advances in meningioma knowledge, the main therapeutic approaches are GTR and radiotherapy [54]. Sharma et al. documented a complex MM case in a 35-year-old female patient with extensive en plaque meningiomas [5]. In this instance, surgical intervention was deemed unfeasible due to the volume and localization of the tumors, which encroached upon critical neurovascular structures. Consequently, the patient was redirected toward radiotherapy, illustrating the necessity for a multidisciplinary approach in treatment planning for patients with extensive disease. Further examples of these complex cases is provided by Ojo et al., who presented three cases of middle-aged female patients with MM, underscoring the clinical variability encountered in this condition [55]. Notably, one of these cases was also categorized as inoperable due to similar constraints observed in the case reported by Sharma et al., highlighting the recurrent theme of surgical limitations in managing multifocal disease [5]. The surgical management of MM often necessitates careful consideration of tumor distribution, neurovascular structure involvement, and potential treatment outcomes. The exploration of alternative therapeutic modalities, such as radiotherapy, becomes imperative in the management strategy of complex MM cases.

It is noteworthy that, until the exhaustive article regarding the management of MM written by Fahlström et al. [7], no comprehensive study had been published on the matter. Thus, specific guidelines are still lacking. Only an overview of current therapeutic management is presently available [7], thanks to these authors.

For the past 15 years, the options available for meningiomatosis, including for high WHO grades, has included fractionated radiosurgery [56] and the use of radioenhancers [57]. These strategies allow for the targeting of multiple lesions and enhance the treatment dose on them while sparing the adjacent healthy brain parenchyma, vascular structures, and radiosensitive cranial nerves. As such, radiosurgical strategies can nowadays offer a remarkable advantage over conventional radiotherapy as first-line radiation options for meningiomatosis. Additionally, gamma knife radiosurgery does not represent a contraindication should the disease recur and a collegial neuro-oncological decision to proceed with a boost of radiotherapy be deemed appropriate at a later stage in patient management [56,57].

Regarding the perioperative complications in MM, challenges relate to tumoral size, localization, and structural characteristics. The main complications associated with meningioma surgeries include postoperative hematoma, infection, brain edema, hydrocephalus, and different medical complications such as pneumonia, renal dysfunction, arrhythmia, deep venous thrombosis, and pulmonary embolism [58,59]. Additionally, there are rare but significant complications that can result in vision loss, including orbital compartment syndrome—which encompasses ophthalmoplegia, vision loss, periorbital edema, exophthalmos, and chemosis— and bilateral central retinal artery occlusion [60,61]. However, our case did not have any perioperative complications related to any of the three performed surgeries.

A brief discussion can be centered on the particularities and uniqueness of our case, including the aggressive growth pattern, the different histopathological gradings of the meningiomas, and the anaplastic transformation at the recurrence of the left convexity frontal meningioma, which was formally identified as WHO grade 1. After the resection in October 2023, the frontal left meningioma was diagnosed as a meningothelial meningioma, WHO grade 1. The meningioma relapsed posteriorly in the left frontoparietal region, with the necessity for neurosurgical intervention in August 2024, only 10 months after the initial resection. The histopathological diagnosis for the frontoparietal recurrence was anaplastic meningioma, WHO grade 3. The number of mitoses significantly increased, from 4 mitoses/10 HPF to 20 mitoses/10 HPF, and the Ki67 proliferation index rose from 25% to 40%, proving the increase in aggressiveness and the change in biological profile. The aggressiveness of the left frontoparietal recurrence was comparable to that of the left frontopolar meningioma, which was diagnosed as anaplastic meningioma WHO grade 3, with more than 20 mitoses/10 HPF and a Ki67 proliferation index of 50%.

Recurrence is not unusual for meningiomas, even for Simpson resections of grade I or II, and can vary from 7 to 20% even in WHO grade 1 meningiomas, which are classically considered benign [53,62]. However, in our case, the rapid recurrence highlights the main particularity of our study. Although the average tumoral growth rate in MM is 0.46 cm^3^/year [7], in our case, the tumor recurred in less than a year, achieving a volume of 77.3 cm^3^, making the case not only a rare finding but also a significant factor that can contribute to developing new guidelines, given the scarce data available.

The growth pattern of meningiomas was studied, and four different statistical models (linear, exponential, linear radial, and Gompertzian) were compared. It is assumed that the Gompertzian model was the best-suited model for meningiomas, as there was an early exponential phase, followed by a linear-like phase and a plateau phase. So, the time needed for the meningeal tumor to double its volume is longer [63,64]. The growth rates of meningiomas differ depending on histological grade, with higher-grade tumors having higher growth rates and shorter tumor doubling times [65,66,67]. However, growth rates may vary widely even among same-grade meningiomas. Some studies tried to compare the growth rate of solitary meningiomas with meningiomas that are part of MM, but no statistically significant differences were found in the growth rates [68]. The tumoral growth dynamics and the potential factors associated with tumoral growth are important for an optimal treatment and follow-up strategy. Despite the fact that specific guidelines for MM management are not available, this is not the case for solitary lesions. For solitary meningiomas, the EANO recommends follow-up MRI imaging according to WHO grades as follows: for WHO grade 1, once a year for 5 years, afterward every 2 years; for WHO grade 2, every 6 months for 5 years, afterward once a year; for WHO grade 3, every 3–6 months indefinitely [14,18,40]. Similarly, for patients with meningiomas where GTR has been achieved, the recommendation is to engage in periodic follow-ups [14]. However, adjuvant radiotherapy is advised in cases where GTR was not completed. In our case, since GTR was accomplished for both the left frontal convexity meningioma and the left frontopolar meningioma, and considering the recurrence of the left frontal convexity meningioma post-resection, the patient was referred to RT as a subsequent treatment strategy.

The intervals between follow-up visits can vary considerably, depending on the extension of resection, the initial size and location of the meningioma, the patient’s age, and neurological status. A consensus does not exist for MM patients, and each case must be treated according to its unique particularities.

Acknowledging the limitations inherent to a single-case study, such as the inability to generalize findings, is of significant importance, especially since there is little data regarding this subject. In this case, genetic testing was not performed, as the patient did not have clinical features of NF2 or other genetic syndromes associated with meningiomas, and genetic testing resources were limited. We classified the case as a sporadic MM because of the lack of risk factors, associated familial meningiomas, history of radiation exposure, or syndromic disease. However, despite the aforementioned limitations, our case report provides further information to the scarce literature regarding this complex medical condition and advocates for the development of international guidelines regarding MM.

## 4. Conclusions

The sudden development of aggressive meningiomatosis, with meningiomas exhibiting different histopathological gradings and histological transformation in recurrent meningiomas, in an adult female patient with no relevant associated medical condition or familial history, encompasses the particularities of this case. MM is a particularly challenging chronic disease due to the presence of multiple symptomatic lesions. Each tumor must be treated as a distinct entity with individual features without minimizing the possible complications associated with MM. A thorough clinical–radiological follow-up is mandatory for the entire lifespan, as recurrences might occur and require repeated treatments. The management of patients with MM is multimodal and complex. It is intended to achieve disease control, to improve quality of life, and to prolong survival. Surgery remains the treatment of choice and should be indicated for symptomatic meningiomas, the general surgical principles being similar to those for solitary meningiomas, and IHC should be performed to obtain important diagnosis and prognostic data. Adjuvant therapies must be considered in the case of aggressive meningiomas or meningiomas located in surgically challenging locations. New, specific international guidelines must be established for MM patients to facilitate therapeutic management and improve outcomes.

## Figures and Tables

**Table 1:** Analysis of the left convexity frontal meningioma, resected in October 2023.

Type	Meningothelial Meningioma WHO-CNS Grade 1
Location	Convexity left frontal mass lesion, attached to the dura
Mitotic index	4 mitoses/10 HPF
Histological description	Meningothelial meningioma with small necrosis areas, rare psammomatous bodies, and mitotic activity of 4 mitoses/10 HPF (in a possible hot spot)
Immunohistochemistry	SSTR2—diffuse positive STAT-6—negative PGR—negative PHH3—positive in 5 mitoses/10 HPF 40× Ki67—proliferation index 25%

**Table 2:** Analysis of left convexity frontopolar meningioma following resection in May 2024.

Type	Anaplastic Meningioma WHO-CNS Grade 3
Location	Convexity left frontopolar mass lesion, attached to the dura
Mitotic index	>20 mitoses/10 HPF
Histological description	Tumoral mesenchymal proliferation with nuclear pleomorphism, important mitotic activity, vascular slits at the level of the stroma, geographic necrosis, and a dense reticulin network
Immunohistochemistry	SSTR2—diffuse positive STAT6—negative PGR—negative PHH3—positive in 20 mitoses/10 HPF 40× Ki67—proliferation index 50%

**Table 3:** Analysis of left convexity frontal recurrence of meningioma following resection in August 2024.

Type	Anaplastic Meningioma WHO-CNS Grade 3
Location	Left convexity frontal mass, attached to the dura
Mitotic index	20 mitoses/10 HPF
Histological description	Anaplastic meningioma with important nuclear atypia, big eosinophilic nucleoli, frequent mitoses, and geographic necrosis
Immunohistochemistry	SSTR2—zonal positive STAT6—negative PGR—negative PHH3—positive in 20 mitoses/10 HPF 40× Ki67—proliferation index 40%

**Table 4:** Description of various genetic syndromes associated with high risk for meningiomatosis.

Genetic Syndromes Associated with Meningiomas	Description	Affected Gene
Neurofibromatosis type 2 (NF-2)	An autosomal dominant genetic syndrome that causes multiple tumors to arise along the CNS	*NF2* gene on the long arm of 22 chromosome coding the tumor suppressor Merlin protein
Cowden syndrome	A rare autosomal dominant syndrome that is characterized by the presence of multiple hamartomas, fibroid tumors, and increased cancer risk	*Phosphate and tensin homolog gene* (PTEN)
MEN 1 (Wermer syndrome)	A rare autosomal dominant endocrine tumor syndrome with high penetrance, characterized by neuroendocrine tumors and also non-endocrine tumors such as CNS tumors, lipomas, and leiomyomas	*Menin gene* (MEN1) on chromosome 11
Werner syndrome (adult progeria)	An autosomal recessive disorder characterized by premature aging, shorter cell lifespan, diabetes mellitus, baldness, cataracts, tumors, and cancer	Gene for Werner disease (*WRN*) on chromosome 8 belonging to the RecQ family
Rubinstein–Taybi syndrome	An autosomal dominant disorder characterized by craniofacial abnormalities, cerebral abnormalities, and cardiac malformations	*CREBBP* gene located on chromosome 16, *EP300* gene located on chromosome 22
Gorlin syndrome (Gorlin–Goltz syndrome, Nevoid basal cell carcinoma syndrome, or basal cell carcinomas BCCs)	An autosomal dominant familial cancer syndrome	A mutation in the *patched gene (PTCH1)* located on chromosome 9
Li–Fraumeni syndrome	An autosomal dominant malignancy predisposition syndrome	*TP53* tumor suppressor gene on chromosome 17

## Data Availability

The data presented in this study are available on request from the corresponding author due to legal reasons.

## References

[1] Ostrom Q.T., Patil N., Cioffi G., Waite K., Kruchko C., Barnholtz-Sloan J.S. (2020). CBTRUS Statistical Report: Primary Brain and Other Central Nervous System Tumors Diagnosed in the United States in 2013–2017. Neuro Oncol..

[2] Maggio I., Franceschi E., Tosoni A., Di Nunno V., Gatto L., Lodi R., Brandes A.A. (2021). Meningioma: Not always a benign tumor. A review of advances in the treatment of meningiomas. CNS Oncol..

[3] Kacemi I.E., Miloudi G. (2021). Multiple meningiomatosis. Pan. Afr. Med. J..

[4] Koech F., Orege J., Ndiangui F., Macharia B., Mbaruku N. (2013). Multiple intracranial meningiomas: A review of the literature and a case report. Case Rep. Surg..

[5] Sharma S., Sharma P., Kumar A. (2021). Diffuse Meningiomatosis Without Neurofibromatosis: A Rare Diagnosis with Atypical Presentation. Indian J. Radiol. Imaging.

[6] Ohla V., Scheiwe C. (2015). Meningiomatosis restricted to the left cerebral hemisphere with acute clinical deterioration: Case presentation and discussion of treatment options. Surg. Neurol. Int..

[7] Fahlström A., Dwivedi S., Drummond K. (2023). Multiple meningiomas: Epidemiology, management, and outcomes. Neurooncol. Adv..

[8] Louis D.N., Perry A., Wesseling P., Brat D.J., Cree I.A., Figarella-Branger D., Hawkins C., Ng H.K., Pfister S.M., Reifenberger G. (2021). The 2021 WHO Classification of Tumors of the Central Nervous System: A summary. Neuro Oncol..

[9] Boulagnon-Rombi C., Fleury C., Fichel C., Lefour S., Bressenot A.M., Gauchotte G. (2017). Immunohistochemical Approach to the Differential Diagnosis of Meningiomas and Their Mimics. J. Neuropathol. Exp. Neurol..

[10] Louis D.N., Perry A., Reifenberger G., Von Deimling A., Figarella-Branger D., Cavenee W.K., Ohgaki H., Wiestler O.D., Kleihues P., Ellison D.W. (2016). The 2016 World Health Organization Classification of Tumors of the Central Nervous System: A summary. Acta Neuropathol..

[11] Pereira B.J.A., de Almeida A.N., de Aguiar P.H.P., Paiva W.S., Teixeira M.J., Marie S.K.N. (2019). Multiple Intracranial Meningiomas: A Case Series and Review of the Literature. World Neurosurg..

[12] Salvati M., Caroli E., Ferrante L., Rocchi G., D’Andrea G., Piccirilli M., Delfini R. (2004). Spontaneous, multiple meningiomas. Zentralbl. Neurochir..

[13] Wang J.Z., Landry A.P., Raleigh D.R., Sahm F., Walsh K.M., Goldbrunner R., Yefet L.S., Tonn J.C., Gui C., Ostrom Q.T. (2024). Meningioma: International Consortium on Meningiomas consensus review on scientific advances and treatment paradigms for clinicians, researchers, and patients. Neuro Oncol..

[14] Goldbrunner R., Stavrinou P., Jenkinson M.D., Sahm F., Mawrin C., Weber D.C., Preusser M., Minniti G., Lund-Johansen M., Lefranc F. (2021). EANO guideline on the diagnosis and management of meningiomas. Neuro Oncol..

[15] Simon M., Gousias K. (2024). Grading meningioma resections: The Simpson classification and beyond. Acta Neurochir..

[16] Tsermoulas G., Turel M.K., Wilcox J.T., Shultz D., Farb R., Zadeh G., Bernstein M. (2018). Management of multiple meningiomas. J. Neurosurg..

[17] Mocker K., Holland H., Ahnert P., Schober R., Bauer M., Kirsten H., Koschny R., Meixensberger J., Krupp W. (2011). Multiple meningioma with different grades of malignancy: Case report with genetic analysis applying single-nucleotide polymorphism array and classical cytogenetics. Pathol. Res. Pract..

[18] Goldbrunner R., Minniti G., Preusser M., Jenkinson M.D., Sallabanda K., Houdart E., von Deimling A., Stavrinou P., Lefranc F., Lund-Johansen M. (2016). EANO guidelines for the diagnosis and treatment of meningiomas. Lancet Oncol..

[19] Alruwaili A.A., De Jesus O. (2025). Meningioma. StatPearls.

[20] Trakolis L., Petridis A.K. (2023). Interdisciplinary Therapeutic Approaches to Atypical and Malignant Meningiomas. Cancers.

[21] Koh Y.-C., Yoo H., Whang G.-C., Kwon O.-K., Park H.-I. (2001). Multiple meningiomas of different pathological features: Case report. J. Clin. Neurosci..

[22] Heinrich B., Hartmann C., Stemmer-Rachamimov A.O., Louis D.N., MacCollin M. (2003). Multiple meningiomas: Investigating the molecular basis of sporadic and familial forms. Int. J. Cancer.

[23] Morrison J.P., Satoh H., Foley J., Horton J.L., Dunnick J.K., Kissling G.E., Malarkey D.E. (2007). N-ethyl-N-nitrosourea (ENU)-induced meningiomatosis and meningioma in p16(INK4a)/p19(ARF) tumor suppressor gene-deficient mice. Toxicol. Pathol,..

[24] Garofola C., Jamal Z., Gross G.P. (2024). Cowden Disease. StatPearls.

[25] Singh G., Mulji N.J., Jialal I. (2024). Multiple Endocrine Neoplasia Type 1. StatPearls.

[26] Chen L., Oshima J. (2002). Werner Syndrome. J. Biomed. Biotechnol..

[27] Stevens C.A., Adam M.P., Feldman J., Mirzaa G., Pagon R., Wallace S., Amemiya A. (2002). Rubinstein-Taybi Syndrome. GeneReviews(^®^).

[28] Spiker A.M., Troxell T., Ramsey M.L. (2024). Gorlin Syndrome. StatPearls.

[29] Schneider K., Zelley K., Nichols K.E., Levine A.S., Garber J., Adam M.P., Feldman J., Mirzaa G., Pagon R., Wallace S., Amemiya A. (1999). Li-Fraumeni Syndrome. GeneReviews(^®^).

[30] Kerr K., Qualmann K., Esquenazi Y., Hagan J., Kim D.H. (2018). Familial Syndromes Involving Meningiomas Provide Mechanistic Insight into Sporadic Disease. Neurosurgery.

[31] Tiwari R., Singh A.K. (2024). Neurofibromatosis Type 2. StatPearls.

[32] Fiorentini E., Giunti L., Di Rita A., Peraio S., Fonte C., Caporalini C., Buccoliero A.M., Censullo M.L., Gori G., Noris A. (2023). SMARCE1-related meningiomas: A clear example of cancer predisposing syndrome. Eur. J. Med. Genet..

[33] Smith M.J., Wallace A.J., Bennett C., Hasselblatt M., Elert-Dobkowska E., Evans L.T., Hickey W.F., van Hoff J., Bauer D., Lee A. (2014). Germline SMARCE1 mutations predispose to both spinal and cranial clear cell meningiomas. J. Pathol..

[34] Munckhof P.v.D., Christiaans I., Kenter S.B., Baas F., Hulsebos T.J.M. (2012). Germline SMARCB1 mutation predisposes to multiple meningiomas and schwannomas with preferential location of cranial meningiomas at the falx cerebri. Neurogenetics.

[35] Wang A.S., Jamshidi A.O., Oh N., Sahyouni R., Nowroozizadeh B., Kim R., Hsu F.P.K., Bota D. (2018). Somatic SMARCB1 Mutation in Sporadic Multiple Meningiomas: Case Report. Front. Neurol..

[36] Singh S.K., Leeds N.E., Ginsberg L.E. (2002). MR imaging of leptomeningeal metastases: Comparison of three sequences. AJNR Am. J. Neuroradiol..

[37] Balm M., Hammack J. (1996). Leptomeningeal carcinomatosis. Presenting features and prognostic factors. Arch. Neurol..

[38] Dong J., Yu M., Miao Y., Shen H., Sui Y., Liu Y., Han L., Li X., Lin M., Guo Y. (2020). Differential Diagnosis of Solitary Fibrous Tumor/Hemangiopericytoma and Angiomatous Meningioma Using Three-Dimensional Magnetic Resonance Imaging Texture Feature Model. Biomed. Res. Int..

[39] Patel M., Nguyen H.S., Doan N., Gelsomino M., Shabani S., Mueller W. (2016). Glioblastoma Mimicking Meningioma: Report of 2 Cases. World Neurosurg..

[40] Nowosielski M., Galldiks N., Iglseder S., Kickingereder P., von Deimling A., Bendszus M., Wick W., Sahm F. (2017). Diagnostic challenges in meningioma. Neuro Oncol..

[41] Moguel A.E.R., Serrano-Rubio A., Soto J.A.G., Suck M.L.T., Roldan-Valadez E., Nunez-Lupaca J.N., Corona-Cedillo R., Mejia A.B., Nathal E. (2024). ntracranial Castleman’s Disease Mimicking Dural-Based Pathologies: A Case Report. Curr. Med. Imaging.

[42] Backer-Grøndahl T., Moen B.H., Torp S.H. (2012). The histopathological spectrum of human meningiomas. Int. J. Clin. Exp. Pathol..

[43] Fritchie K.J., Jin L., Rubin B.P., Burger P.C., Jenkins S.M., Barthelmeß S., Moskalev E.A., Haller F., Oliveira A.M., Giannini C. (2016). NAB2-STAT6 Gene Fusion in Meningeal Hemangiopericytoma and Solitary Fibrous Tumor. J. Neuropathol. Exp. Neurol..

[44] Mosquera J.M., Fletcher C.D. (2009). Expanding the spectrum of malignant progression in solitary fibrous tumors: A study of 8 cases with a discrete anaplastic component--is this dedifferentiated SFT?. Am. J. Surg. Pathol..

[45] Saranraj M.K., Shanmugam S., Raiyani Y., Jacob A., Ghosh S. (2024). Multiple Meningiomas with Different Histological Patterns in the Same Patient: Do They Exist? A Case Report and Literature Review. Indian J. Neurosurg..

[46] Liu Y., Song D.P., Wang T. (2017). Meningiomas with different histological grade in the same patient: Case report. Medicine.

[47] Mizrachi M., Hartley B., Saleem S., Hintz E., Ziemba Y., Li J., Goenka A., Schulder M. (2024). Ki-67 index as a predictive marker of meningioma recurrence following surgical resection. J. Clin. Neurosci..

[48] Fountain D.M., Young A.M.H., Santarius T. (2020). Malignant meningiomas. Handb. Clin. Neurol..

[49] Patel B., Desai R., Pugazenthi S., Butt O.H., Huang J., Kim A.H. (2022). IIdentification and Management of Aggressive Meningiomas. Front. Oncol..

[50] Chun S.-W., Kim K.M., Kim M.-S., Kang H., Dho Y.-S., Seo Y., Kim J.W., Kim Y.H., Park C.-K. (2021). Adjuvant radiotherapy versus observation following gross total resection for atypical meningioma: A systematic review and meta-analysis. Radiat. Oncol..

[51] Hasan S., Young M., Albert T., Shah A.H., Okoye C., Bregy A., Lo S.S., Ishkanian F., Komotar R.J. (2015). The role of adjuvant radiotherapy after gross total resection of atypical meningiomas. World Neurosurg..

[52] Mair M.J., Berghoff A.S., Brastianos P.K., Preusser M. (2023). Emerging systemic treatment options in meningioma. J. Neurooncol..

[53] Saraf S., McCarthy B.J., Villano J.L. (2011). Update on meningiomas. Oncologist.

[54] Chen W.C., Perlow H.K., Choudhury A., Nguyen M.P., Mirchia K., Youngblood M.W., Lucas C.H.G., Palmer J.D., Magill S.T., Raleigh D.R. (2022). Radiotherapy for meningiomas. J. Neurooncol..

[55] Ojo A., Fynn E. (2006). Multiple meningiomas. S. Afr. J. Radiol..

[56] Ganau M., Foroni R.I., Gerosa M., Zivelonghi E., Longhi M., Nicolato A. (2014). Radiosurgical options in neuro-oncology: A review on current tenets and future opportunities. Part I: Therapeutic strategies. Tumori.

[57] Ganau M., Foroni R.I., Gerosa M., Ricciardi G.K., Longhi M., Nicolato A. (2015). Radiosurgical options in neuro-oncology: A review on current tenets and future opportunities. Part II: Adjuvant radiobiological tools. Tumori.

[58] Lemée J.-M., Corniola M.V., Da Broi M., Schaller K., Meling T.R. (2019). Early Postoperative Complications in Meningioma: Predictive Factors and Impact on Outcome. World Neurosurg..

[59] Sughrue M.E., Rutkowski M.J., Shangari G., Chang H.Q., Parsa A.T., Berger M.S., McDermott M.W. (2011). Risk factors for the development of serious medical complications after resection of meningiomas. Clinical article. J. Neurosurg..

[60] Zilka T., Harag T., Illes R., Smrcka M., Lucia K. (2023). Sudden onset of complete ophthalmoplegia and blindness after resection of large frontal convexity meningioma: Case report. Interdiscip. Neurosurg..

[61] Lizana J., Reinoso C.M.D., Aliaga N., Marani W., Montemurro N. (2021). Bilateral central retinal artery occlusion: An exceptional complication after frontal parasagittal meningioma resection. Surg. Neurol. Int..

[62] Pettersson-Segerlind J., Orrego A., Lönn S., Mathiesen T. (2011). Long-Term 25-Year Follow-up of Surgically Treated Parasagittal Meningiomas. World Neurosurg..

[63] Strand P.S., Wågø K.J., Pedersen A., Reinertsen I., Nälsund O., Jakola A.S., Bouget D., Hosainey S.A.M., Sagberg L.M., Vanel J. (2023). Growth dynamics of untreated meningiomas. Neuro-Oncol. Adv..

[64] Nakasu S., Nakasu Y., Fukami T., Jito J., Nozaki K. (2011). Growth curve analysis of asymptomatic and symptomatic meningiomas. J. Neurooncol..

[65] Jääskeläinen J., Haltia M., Laasonen E., Wahlström T., Valtonen S. (1985). The growth rate of intracranial meningiomas and its relation to histology. An analysis of 43 patients. Surg. Neurol..

[66] Nakamura M., Roser F., Michel J., Jacobs C., Samii M. (2005). Volumetric analysis of the growth rate of incompletely resected intracranial meningiomas. Zentralbl. Neurochir..

[67] Fountain D.M., Soon W.C., Matys T., Guilfoyle M.R., Kirollos R., Santarius T. (2017). Volumetric growth rates of meningioma and its correlation with histological diagnosis and clinical outcome: A systematic review. Acta Neurochir..

[68] Wong R., Wong A., Vick N., Farhat H. (2013). Natural history of multiple meningiomas. Surg. Neurol. Int..

